# Isolation and mass spectrometry based hydroxyproline mapping of type II collagen derived from *Capra hircus* ear cartilage

**DOI:** 10.1038/s42003-019-0394-6

**Published:** 2019-04-29

**Authors:** Priti Prasanna Maity, Debabrata Dutta, Sayan Ganguly, Kausik Kapat, Krishna Dixit, Amit Roy Chowdhury, Ramapati Samanta, Narayan Chandra Das, Pallab Datta, Amit Kumar Das, Santanu Dhara

**Affiliations:** 1 School of Medical Science and Technology, Indian Institute of Technology Kharagpur, Kharagpur, 721302 India; 2 Centre for Healthcare Science and Technology, Indian Institute of Engineering Science and Technology, Shibpur, Howrah, 711103 India; 3 Department of Biotechnology, Indian Institute of Technology Kharagpur, Kharagpur, 721302 India; 4 Rubber Technology Centre, Indian Institute of Technology Kharagpur, Kharagpur, 721302 India

**Keywords:** Sequencing, Stem cells, Proteomic analysis, Isolation, separation and purification, Mass spectrometry

## Abstract

Collagen II (COLII), the most abundant protein in vertebrates, helps maintain the structural and functional integrity of cartilage. Delivery of COLII from animal sources could improve cartilage regeneration therapies. Here we show that COLII can be purified from the Capra ear cartilage, a commonly available bio-waste product, with a high yield. MALDI-MS/MS analysis evidenced post-translational modifications of the signature triplet, Glycine-Proline-Hydroxyproline (G-P-Hyp), in alpha chain of isolated COLII (COLIIA1). Additionally, thirty-two peptides containing 59 Hyp residues and a few G-X-Y triplets with positional alterations of Hyp in COLIIA1 are also identified. Furthermore, we show that an injectable hydrogel formulation containing the isolated COLII facilitates chondrogenic differentiation towards cartilage regeneration. These findings show that COLII can be isolated from Capra ear cartilage and that positional alteration of Hyp in its structural motif, as detected by newly developed mass spectrometric method, might be an early marker of cartilage disorder.

## Abstract (web summary)

Priti Prasanna Maity et al. develop a method for high-yield isolation of type II collagen from the *Capra hircus* ear cartilage, a commonly available biowaste product. Using mass spectrometry they mapped the positions of hydroxyproline within the collagen II alpha chain; this methodology facilitates isolation and characterization of this biomedical resource.

## Introduction

Collagen is the most abundant protein playing an important role in maintaining the structural and functional integrity of the tissues through their self-interaction. Collagen triple-helix with ~300 repetitive sequences of Gly-X-Y (where X is often proline and Y is hydroxyproline)^1^ is usually identified from nucleotide sequences. Such positional assignment of proline and hydroxyproline (Hyp) ensures stabilization of the triple helix structure through water-bridged intermolecular hydrogen bonding^2^. It is reported that Hyp in collagen is derived from the ascorbic acid-dependent post-translational modification (PTM) of proline residues. This PTM is essential for structural stability of native collagen to minimize pathological dysfunction^1^. COLII, the major protein component in cartilage, consisting of three alpha I (COLIIA1) chains, is enriched with hydroxylation of proline and lysine as well as glycosylation of lysine to maintain its secondary and tertiary structures^3^. Although the primary structure of collagen molecules is usually identified from their nucleotide sequences, there is enormous conflict on their amino acid sequences owing to the changes during PTM, especially hydroxylation of lysine and proline residues^3,4^. Hyp has an important role in supramolecular fibril network formation of COLII embedded in extracellular matrix^2,5^. Owing to expressional alterations of hydroxylation of proline, lysine, and glycosylation of hydroxylysine during PTM, the resultant COLII may act as auto-antigen epitope in cartilage tissues causing auto-immune response. Accordingly, PTM is an important biochemical fingerprint for detection and verification of collagen functions during modulation of key cellular signaling pathways^3^. Notably, lack of proline hydroxylation in COLIIA1 during PTM promotes dysfunctional collagen assembly in extracellular matrix^2^. Therefore, identification and mapping of Hyp position in signature motif plays an important role to correlate molecular alteration of COLIIA1 with progression of arthritis^6–8^.

Here we show the isolation of COLII from *Capra* ear cartilage through bio-waste recycling. Post-translationally modified proline residues (i.e., Hyp PTM) could be identified and mapped through tandem mass spectrometry using MASCOT database^9^. Hyp mapping is performed to validate its sequential alteration in Gly–X–Y structural motif in COLIIA1. This identification may contribute to the prediction of dysfunctional collagen leading to arthritis as well as molecular identification of COLII from other sources. Moreover, the isolated COLII is transformed into hydrogel along with Pluronic F127 in combination with *Capra* adipose tissue-derived stem cells (ADMSCs) towards differentiation of chondrogenic lineage in vitro.

## Results

### Isolation and identification of COLII from Capra ear cartilage

**Fig. 1:**
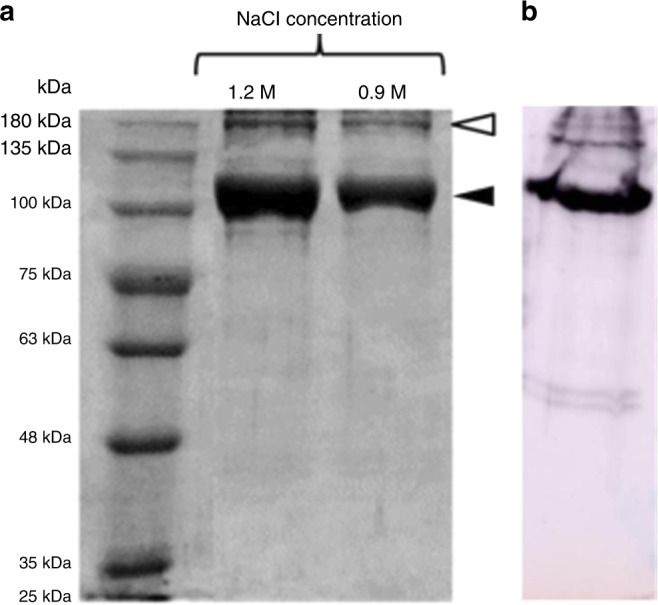
Identification of purified COLII by SDS-PAGE and western blot analysis. **a** SDS-PAGE showing the isolated COLII with two prominent bands where filled arrow indicating α chain and open arrow indicating β chain and **b** α chain was identified using anti-COLII antibody using western blot analysis

Isolation of COLII was optimized using 0.1% (w/v) pepsin digestion followed by 1.2 M NaCl precipitation to produce yield of ∼55% (on dry weight basis) (Supplementary Fig. 1). Hyp content was estimated to be ∼118 mg/g of COLII^10,11^. SDS-PAGE profile of (Fig. 1a) isolated COLII showed two prominent bands at 122 kDa and 200 kDa for α chain and β sheet, respectively. Purity of COLII was found to be higher in case of 1.2 M NaCl precipitation as compared to that of 0.9 M. Moreover, isolated COLII was identified using anti-COLII antibody (Abcam, USA) (Fig. 1b) and absence of elastin in COLII was confirmed by anti-elastin antibody (Abcam, USA) using western blot analysis (Supplementary Fig. 2).

### Physico-chemical characterization of COLII Fourier Transforms Infrared (FTIR) spectroscopy

**Fig. 2:**
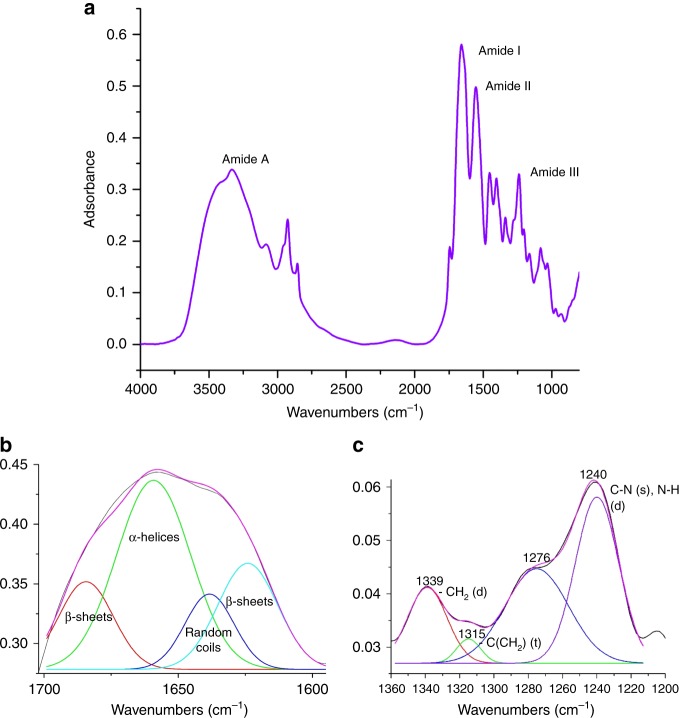
FTIR analysis of COLII. **a** FTIR spectrum of COLII; deconvoluted spectra of **b** Amide I and **c** Amide III showing characteristic signature of COLII

FTIR spectrum represents major absorption bands observed at 3336 cm^−1^, 1658 cm^−1^, 1555 cm^−1^, and 1240 cm^−1^ were attributed to amide A, amide I, amide II and amide III, respectively, for purified COLII (Fig. 2a). The spectrum of Amide I band, after deconvolution (Fig. 2b), reveals 1624 cm^−1^, 1638 cm^−1^, 1659 cm^−1^, 1684 cm^−1^ for β-sheets, random coils, α-helices and β-sheets^12^, respectively. Similarly deconvolution of Amide III (Fig. 2c) reveals peaks at 1200–1350 cm^−1^ with 1339 cm^−1^, 1315 cm^−1^, 1240 cm^−1^ representing CH_2_ deformation, –C(CH_2_) twisting and CN stretching (also NH deformation), respectively^13,14^.

### Circular dichroism (CD) spectra of COLII

**Fig. 3:**
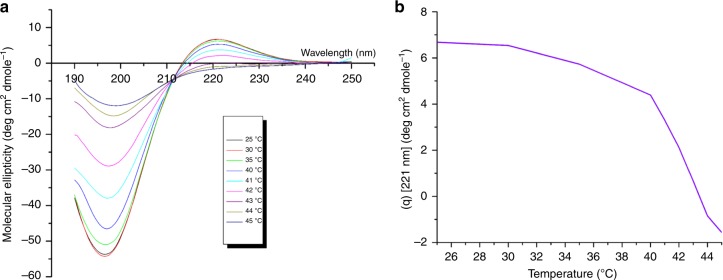
CD analysis of purified COLII. **a** CD spectra of COLII obtained at different temperatures (25–45 °C) and **b** at 221 nm showing denaturation temperature at 43 °C

CD spectra of COLII in a range of 25–45 °C is shown in Fig. 3a. The spectra show a dichroic signal maximum at 221 nm (positive band), minimum at 198 nm (negative band) with consistent cross over point (zero rotation) at about 212 nm ensuring characteristic triple helical conformation of COLII^15,16^. Figure 3b represents corresponding mean molar ellipticity, [*θ*]_221_, as function of temperature. Values of [*θ*]_221_ decreased with the increase of temperature due to denaturation of collagen triple helical structure^17^. Denaturation temperature (*T*_d_) was measured to be 43 °C. The percentage of intactness and denaturation of COLII at different temperatures^16^ have been shown in Supplementary Table 1.

### Field emission scanning electron microscopy (FESEM) of COLII

**Fig. 4:**
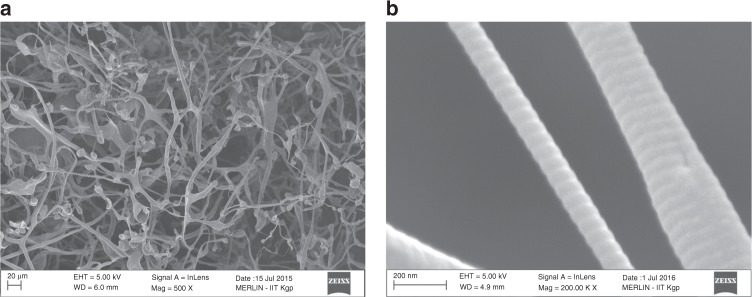
FESEM images of COL II. **a** Showing the overlapping fibrilar structure and **b** characteristic D-spacing value of ~67 nm

FESEM images depicted the overlapping COLII fibers with characteristic D-banding pattern (Fig. 4a, b). In collagen fibers, tropo-collagens were aligned in a parallel manner while maintaining the gap overlapping pattern. Results showed alternate light/dark-band pattern, referring to D spacing of collagen associated with regular arrangement of the molecules. The calculated D-spacing value was ∼67 nm, similar to the values reported elsewhere^17^.

### Amino acid profiling of COLII

Amino acid profiling of COLII was carried out by HPLC analysis and the results showed higher contents of G, P, and Hyp residues (i.e., 302, 99, and 118 residues per 1000 amino acids residues, respectively) compared to the contents of tyrosine, cysteine, histidine and methionine residues (i.e., 5, 20, 5, and 10 residues, respectively) (Supplementary Table 2). Similar results for cartilage COLII were reported elsewhere^18^.

### Proteomic characterization of COLII

**Fig. 5:**
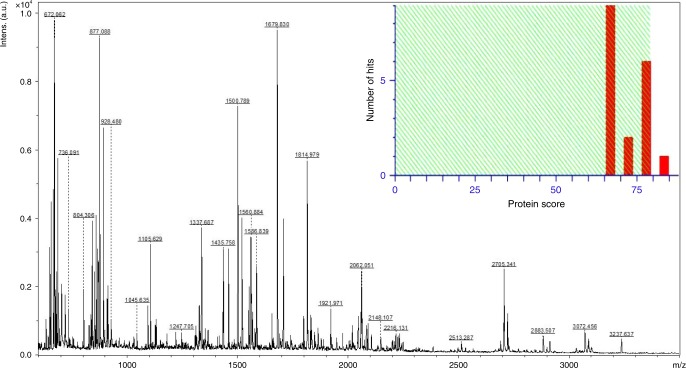
Mass spectrometric analysis of purified COLII. MALDI-MS spectrum of tryptic peptides derived from COLIIA1 protein band and identification with MASCOT histogram showing significant (*p* < 0.05) MASCOT score (82) for COLIIA1 of *Bos taurus*

Peptide extraction and MALDI MS analysis: MALDI-TOF/TOF analysis of COLIIA1 band (~122 kDa) from SDS-PAGE exhibited peptide fragments of COLIIA1 after tryptic digestion (Fig. 5; Supplementary Table 3). Individual peptides were further used for MS/MS analysis by adopting reported protocol published elsewhere^19^.

Peptide mass fingerprinting (PMF) of COLIIA1 for PTM analysis: The full length primary sequence of COLIIA1 of *Capra hircus* was unavailable in MASCOT database. Therefore, our evaluation of COLIIA1 sequence was based on amino acid sequence of *Bos taurus* (Uni-Prot accession number: P02459) and *Homo sapiens* (Uni-Prot accession number: P02458), which are closely related to *Capra hircus*. Primary amino acid sequence of *Capra hircus* COLIIA1 (KEGG entry: 100860743) derived from genome sequence^20^ showed 98% identity with *Bos taurus* COLIIA1 (Supplementary Fig. 3). The obtained peptide fragments ranging between 700 and 3500 Dalton from in-gel digestion showed ~30% coverage in full length sequence of *Bos taurus* COLIIA1 (Supplementary Fig. 4). To investigate variable modification of proline oxidation, SwissProt database searching option was adopted for detection of Hyp PTM in fragments peptides of COLIIA1 through addition of 16 Da to proline residues depicting Hyp. MASCOT identified 32 peptides (Table 1) where 59 Hyp PTM were present in COLIIA1.

**Table 1:** COLIIA1 peptides of *Capra hircus* containing Hyp residues identified by peptide mass fingerprinting analysis

*m*/*z*	Range	Amino acid sequences	No. of P residue (s)	No. of Hyp residue (s)
852.4903	267–275	GPPGPQGAR	3	1
1976.0676	267–287	GPPGPQGARGFPGTPGLPGVK	6	2
1584.8541	420–437	GSAGAPGIAGAPGFPGPR	4	3
1353.6891	495–509	GEPGGAGPAGPPGER	4	3
1921.9713	495–515	GEPGGAGPAGPPGERGAPGNR	5	4
1366.6988	528–542	GAPGERGPSGLAGPK	3	1
1706.8428	543–560	GANGDPGRPGEPGLPGAR	4	2
1326.7555	561–574	GLTGRPGDAGPQGK	2	1
2045.0664	575–596	VGPSGAPGEDGRPGPPGPQGAR	6	2
2061.0739	575–596	VGPSGAPGEDGRPGPPGPQGAR	6	3
853.4861	621–629	GLPGAPGLR	2	1
869.4979	621–629	GLPGAPGLR	2	2
1337.6867	621–634	GLPGAPGLRGLPGK	3	3
2148.1069	630–653	GLPGKDGETGAAGPPGPAGPAGER	5	2
1679.8304	635–653	DGETGAAGPPGPAGPAGER	4	1
678.3628	702–707	GFPGER	1	1
1128.5859	720–731	GLPGTPGTDGPK	3	2
2913.5118	732–764	GAAGPAGPPGAQGPPGLQGMPGERGAAGIAGPK	7	4
2106.0665	825–848	GETGPPGPAGFAGPPGADGQPGAK	6	1
1500.7894	888–904	GAQGPPGATGFPGAAGR	3	2
912.5034	981–989	GIVGLPGQR	1	1
1328.7270	993–1006	GFPGLPGPSGEPGK	4	2
2225.1735	993–1016	GFPGLPGPSGEPGKQGAPGASGDR	5	2
1798.9796	1017–1036	GPPGPVGPPGLTGPAGEPGR	7	2
1814.9789	1017–1036	GPPGPVGPPGLTGPAGEPGR	7	3
2513.2866	1056–1084	GDRGETGAVGAPGAPGPPGSPGPAGPIGK	7	2
1567.8021	1089–1106	GEAGAQGPMGPAGPAGAR	3	1
928.4796	1107–1115	GMPGPQGPR	3	2
2705.3415	1134–1163	GFTGLQGLPGPPGPSGDQGASGPAGPSGPR	7	2
2721.3574	1134–1163	GFTGLQGLPGPPGPSGDQGASGPAGPSGPR	7	3
1519.8262	1175–1190	DGANGIPGPIGPPGPR	5	3
1551.7982	1175–1190	DGANGIPGPIGPPGPR	5	5

**Fig. 6:**
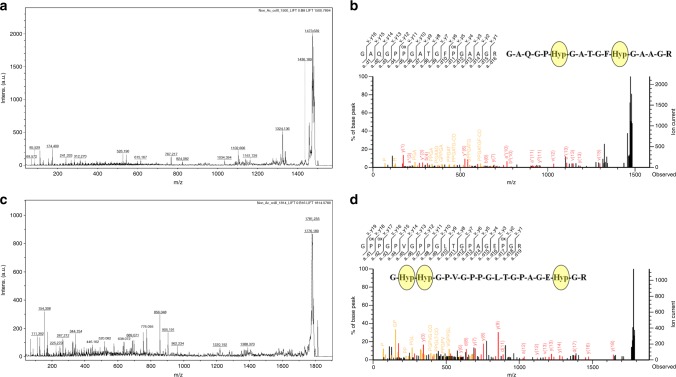
Hyp-PTM mapping of COLIIA1 peptides. MALDI MS/MS spectra and fragment ion annotations of **a**, **b** 1500 Da and **c**, **d** 1814 Da peptides showing specific position of Hyp PTM in the sequences. “P_ox_” represents the Hyp residues

Site specific characterization of Hyp PTM in G-X-Y triplets: MALDI tandem mass spectrometry (MS-MS) identified fragmented ions derived from tryptic peptides of COLIIA1 for site specific characterization of Hyp PTM. Therefore, 1500 and 1814 Da peptides were selected for MS/MS fragmentation shown in Fig. 6a, c. Fragment ion analysis of 1500 and 1814 Da peptides identified Hyp position at 1018, 1019 number amino acid of G-Hyp-Hyp, 893 of G-P-Hyp and 899, 1034 of G-X-Hyp, the G-X-Y triplets present in COLIIA1 primary sequence (Fig. 6b, d; Table 1; Supplementary Fig. 4). Hyp positions were annotated by fragmented ions (b and y ions) of 1500 and 1814 Da peptides (Fig. 6b, d).

### Assessment of chondrogenic potential of COLII derived injectable hydrogel

**Fig. 7:**
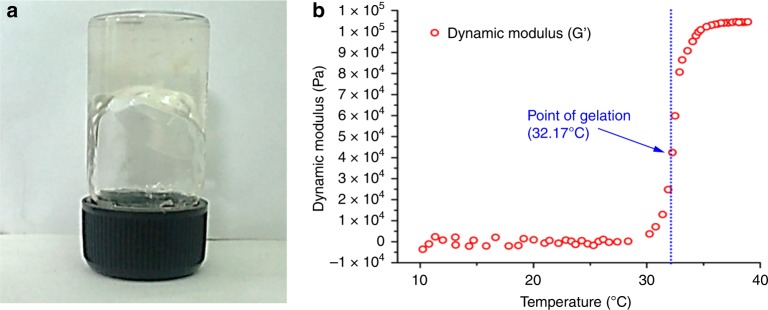
Assessment of gelation temperature of CP hydrogel. **a** Gelation temperature was found to be 32 °C using vial inversion test and **b** change in storage modulus under temperature sweep oscillatory rheological measurement at constant frequency of 1 Hz

Preparation of thermo-reversible hydrogel (CP): Thermo-responsive hydrogel containing COLII and Pluronic F127 copolymer was prepared at 4 °C. The gelation was evidenced within 30 s after reaching the temperature at ~32 °C. Gelation was visualized by vial-inversion method as shown in Fig. 7a. The temperature sweep experiment evidenced sharp change in storage modulus (*Ǵ*) as shown in Fig. 7b. The 10^2^ fold increase in *Ǵ* value supported the increase in storage modulus thereby gel formation. At below-ambient temperature, the hydrogel was mobile enough and showed minimal storage modulus in the sub-Pascal range confirming the Newtonian fluidity^21^. After increasing temperature, the system responded drastically by rapid rise in storage modulus (*Ǵ*) value. This point is implied as the “critical gel point” at ~32.17 °C.

Isolation and identification of ADMSCs from Capra hircus: The isolated ADMSCs were identified by immune-fluorescence (IF) staining and real time PCR (RT-PCR). After 3rd passage, isolated cells from Capra adipose tissue showed distinct expression of surface marker, CD 44 along with the absence of CD 31 depicting ADMSCs characteristics. Positive expression of CD 90, CD 73, CD 105, and negative expression of CD 45 surface marker related genes were confirmed by RT-PCR analysis as well (Supplementary Fig. 5). Trilineage (adipogenic, osteogenic and chondrogenic) differentiation potential of isolated ADMSCs were also assessed after 21 days of differentiation studies. After adipogenic differentiation, accumulation of lipid droplets in cell cytoplasm was observed through Oil Red O staining, whereas migration of cells in closer contact forming occasional clumps as well as positive in Alcian blue staining were witnessed after chondrogenic differentiation. When supplemented with the osteogenic medium, cells deposited extracellular calcium crystals, which were detected through Alizarin Red S staining. However, such trilineage differentiation characteristics were absent in control supplemented with regular medium (Supplementary Fig. 6)^22^.

**Fig. 8:**
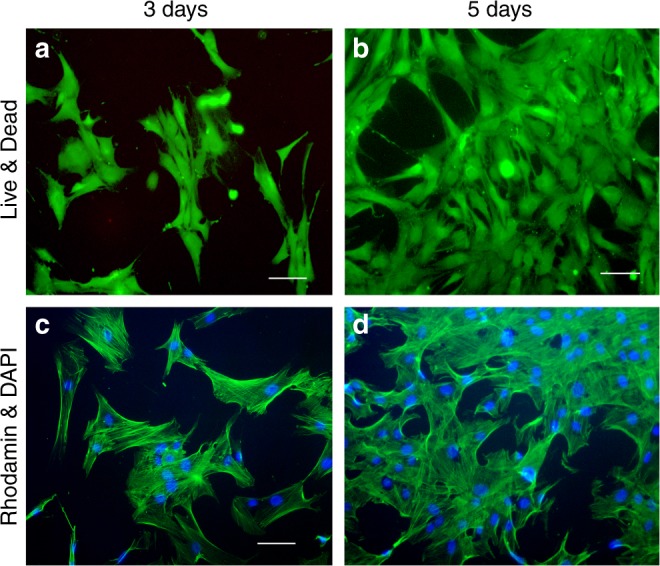
Assessment of cytotoxicity and cell proliferation of CP hydrogel. Adhesion, viability and morphological characteristics of ADMSCs seeded on CP hydrogel showed insignificant number of dead cells with unaltered cellular morphology through **a**, **b** Live-Dead assay and **c**, **d** Rhodamine–DAPI staining (Pseudo Color). Scale bar = 100 µm

Assessment of cytotoxicity and cell proliferation using CP hydrogel: Adhesion, viability and morphological characteristics of ADMSCs seeded on CP hydrogel were analysed by live-dead and Rhodamin-DAPI staining. Figure 8 shows relatively higher cell adhesion and proliferation on 5d as compared to 3d culture indicating cytocompatible nature of the hydrogel. Presence of insignificant dead cells after live-dead staining also signifies its non-cytotoxic nature. After 3d and 5d culture, Rhodamine-DAPI staining revealed similar morphology of ADMSCs within CP hydrogel as compared to TCP.

**Fig. 9:**
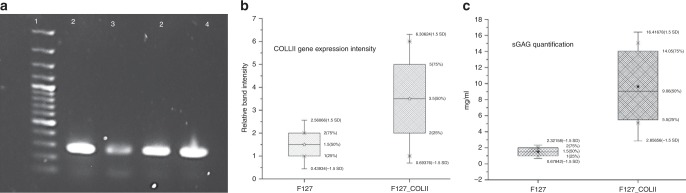
Expression of cartilage related genes (COLII) on F127 and F127_COLII hydrogels seeded with Capra ADMSCs. **a** RT-PCR profiles (lane 1–4 100 bp Ladder, GAPDH, F-127 and COLII, respectively). **b** Relative band intensity and **c** quantitative estimation of sGAGs after 21 days of ADMSCs differentiation culture

Gene expression analysis and sulfated glycosaminoglycan (sGAG) quantification: Chondrogenic potential of CP hydrogel was evaluated by analysing cartilage related gene (COLII) expression using RT-PCR. Expression of COLII was upregulated for cells grown within CP hydrogel as compared to that of F127 hydrogel (*P* ≤ 0.0001) (Fig. 9a, b). Further, accumulation of sGAG was significantly higher with CP hydrogel (13.63 ± 1.35 mg) in comparison with control (5.64 ± 0.62 mg) (*P* < 0.0008) (Fig. 9c).

## Discussion

Cartilage regeneration using COLII by oral nutrient supplementation or using injectable gel based delivery vehicle could be a cutting edge alternative therapeutic approach. To meet increasing demands of COLII, various methods are being explored by researchers to isolate this protein from different sources. *Capra* ear cartilage is one such bio-waste resources, which is not remarkably explored for isolation of COLII.

Here we show a simple, rapid isolation process of COLII in a cost effective way. The process demonstrates a simple route to separate skin from *Capra* ear as an alternative towards easy isolation of cartilage using hypertonic solution following 0.04% pepsin digestion at 37 °C for 72 h, a novel addition to the existing process. Pepsin (0.1%) based tissue digestion is performed at room temperature to increase the activity of pepsin resulting in considerable higher yield (≥55%)^23^. This enzymatic digestion also enabled removal of N-terminal and C-terminal of non-helical COLII chain (telopeptide region) through breakdown of intermolecular crosslinks usually formed through aldol condensation^10,11^. Removal of telopeptide (N-terminal and C-terminal) and breakdown of secondary structures are necessary to reduce antigenicity, while increasing biocompatibility and biodegradability^11^. The existing process for isolating COLII from other resources reported maximum yield of 9–55% (on dry weight basis) using 1% pepsin digestion followed by precipitation with 0.9 M NaCl in 5–7 days. The present study demonstrates ~55% yield of COLII from *Capra* ear cartilage using 1/10th dose of pepsin followed by precipitation with 1.2 M NaCl in 48 h, notably reducing the processing cost^16,18,24^.

FTIR study revealed changes in the secondary structure of isolated COLII. The shift of amide A spectral region to marginally lower value (3336 cm^−1^) as compared to that of the other proteins (3400–3440 cm^−1^) indicates formation of H-bonds between N–H groups and other groups, resulting in a stable triple helix structure^25^. The intense peak of amide I (1658 cm^−1^) associated with amide residues indicates that intermolecular crosslinks and β turns in isolated COLII remained unchanged, similar to that of native cartilage^26^. On the other hand, random coil state was shifted towards lower wave number indicating slight denaturation during isolation process at room temperature^13^. Presence of amide II band (1553 cm^−1^) indicates retention of the triple helical structure of the isolated protein^26^. Amide III bands at 1200–1350 cm^−1^ is known to be the fingerprint region of collagen molecule. Changes in this region is directly related to changes in the native tripeptide (Gly-Pro-Hyp)_n_ sequences of collgen and endorsed to different identification of a same molecule^27^. The deconvoluted peaks in this region strongly suggest that the signature sequence of COLII (Gly-Pro-Hyp) remained unaffected in the isolated protein, which was in agreement to the result reported by Bachmann et al.^27^. This finding includes deconvolution of specific band recommends that the extracted protein of *Capra* ear cartilage is COLII and retained native intermolecular crosslinking during isolation and purification process.

CD is an essential tool for rapid evaluation of secondary structure and protein folding through unequal absorption of left-handed and right-handed circularly polarized light while passing through prism/filter with the sinusoidal oscillation of electric field in a single plane. Herein, the CD was effectively utilized to study the extent of denaturation of the COLII, which was isolated by a temperature dependent process. In proteins, chromophores in polypeptide backbone are aligned in arrays and their optical spectra has evidenced multiple transitions due to exciton interactions. Being a triple helix structure, COLII had three distinct CD transitions at 221 nm (positive band), 198 nm (negative band), and 212 nm (crossover point or zero rotation) confirming its secondary structure. The spectral analysis also revealed deformation of COLII triple helix structure along with shift as well as decrease in magnitude of the negative band (198 nm) with a parallel loss of the positive band (221 nm) against temperature sweep during CD measurement^28^. This triple helix structure transformed into a random coil when the temperature was increased above denaturation temperature (*T*_d_). Percentage intactness, calculated from the predicted secondary structure of COLII against increasing temperature, started reducing at 41 °C and reached to the maximum value at 43 °C^15^, which was designated as denaturation temperature (*T*_d_) of this protein. Therefore, the study established the optimized conditions for isolation of COLII from *Capra* ear cartilage without disrupting its triple helical structure.

Changes of COLII microstructure associated with its functional properties were explored by FESEM. The isolated COLII via acetic acid/pepsin based digestion process, exhibited fibrillar and multi-layered structure similar to native structures as reported elsewhere^16,18^. The tropo-collagens in collagen fibers are aligned in a parallel manner while maintaining the repeating gap overlapping pattern. This gap overlapping pattern is referred to D-spacing (∼67 nm length) associated with regular arrangement of collagen microfibrils^17^.

Collagen molecule consists of a unique triple-helical structure with three left-handed polyproline II like chains supercoiled in a right-hand manner along a common axis. To confirm this structure, the glycine residues at every third position should be present in the amino acid sequence. Therefore, amino acid profiling of COLII indicates presence of high content of glycine, hydroxyproline, and proline with small amounts of tyrosine, histidine and methionine per 1000 amino acids residues. The high glycine content in α-chain of COLII signifies its presence in every third residue in the sequence, except in first 14 amino acid residues of the N-terminus and first 10 amino acid residues of the C-terminus^18^. Presence of high content of other amino acids (proline and hydroxyproline) in α-chain contributed towards formation of planar peptide bonds. These assumptions indicates to the construction of correct model of *Capra* ear cartilage derived COLII as a (Gly-X-Y)_n_ pattern. Indeed, it is the most commonly reported triplet in collagen α chain space^16^.

Peptide mapping of COLII obtained through precipitation in different NaCl concentration exhibited similar migration bands, which were composed of three identical α-chains[α(II)_3_] with molecular weight 122 kDa reported elsewhere^18^. Bands obtained in SDS-PAGE and subsequent western blot analysis using anti-collagen II antibody (Abcam, USA) indicated high purity of isolated COL II^18,16,29^. Furthermore, western blot analysis using anti-elastin antibody (Abcam, USA) confirmed absence of any elastin protein in the isolated COLII from *Capra* ear cartilage. The result indicates that NaOH pre-treatment for *Capra* ear cartilage is necessary for the removal of non-collagenous protein from tissue^30^.

The predicted and partial genome sequences of *Capra hircus* and its collagen gene isoforms are available in gene database. Till date, *Capra hircus* COLIIA1 protein sequence is partially available in the SwissProt; but its primary sequence is available in the KEGG organism database (http://www.genome.jp) from genome annotation^20^. Herein, we attempted to identify COLII especially the α1 chain of *Capra hircus* using primary sequence present in KEGG database. After enzymatic digestion, an extensive number of COLIIA1 peptides are matched with COLIIA1 of *Capra hircus* (KEGG Ref. No. 100860743), *Bos taurus* (SwissProt Ref. No. 407142) and *Homo sapiens* (SwissProt Ref. No. 1280). Number of amino acids present in a single peptide is about 4–15. At molecular level analysis of COLIIA1, the well-known triplet structure (Gly-X-Y) of this protein is also identified with identical positions of X and Y for proline and Hyp, respectively. The presence of Hyp in Y position ensures stabilization of triple helix structure of COLIIA1. However, alterations of amino acids in these positions associated with PTM (such as hydroxylation of proline, lysine and glycosylation of hydroxylysine) serve as epitopes behaving like auto-antigen in cartilage tissues triggering immune system leading to autoimmune disorders. Additionally, lack of proline hydroxylation (Hyp) as an important PTM in COLIIA1 leading to a functional abnormality of collagen found in cartilage extracellular matrix^1^. It may be due to numerous functional relationships with other biomolecules associated with developmental biology. Therefore, identification and mapping of Hyp and its position in the signature triplet sequence (Gly-X-Y) are very important for molecular identification of COLIIA1 of *Capra hircus*. Here, we report the peptide mass fingerprinting (PMF) identification and tandem mass spectrometry-based mapping of Hyp PTM in COLIIA1 from *Capra hircus* using MASCOT database. Since the database did not contain information related to *Capra hircus* COLIIA1 sequence, our identification was primarily based on homologous COLIIA1 sequence from *Bos taurus* and *Homo sapiens* (above 98% identity). Findings include identification of new triplet Gly-F-Hyp in *Capra hircus* COLIIA1 and well known Gly-X-Y triplet with alteration of Hyp at the X position. This positional alteration of Hyp or Proline in Gly-X-Y triplet may be a valuable motif for prediction of functional abnormality of cartilage in collagen assembly concerning osteo-rheumatoid arthritis^6^. These motifs are important for molecular identification and characterization of COLII from other sources as well.

Isolated COLII was blended with Pluronic F127 for preparation of COLII-Pluronic (CP) hydrogel. Pluronic F127 is non-toxic FDA approved poly (ethylene oxide)/poly(propylene oxide)/poly(ethylene oxide) (PEO-PPO-PEO) triblock copolymers. Owing to non-toxicity and thermal gelation property, this polymer is considered as an excellent drug and cell delivery vehicle. Aqueous solution of this polymer undergoes sol-to-gel transition above a certain lower critical gelation temperature owing to amphiphilic character of poly (propylene oxide) blocks which form a volume-filling cubic array of micelles at elevated temperatures. Isolated COLII was incorporated in Pluronic F127 gel base, where COLII is an important protein for cartilage regeneration and F127 maintaining thermo-reversible properties of the resultant hydrogel. Therefore, such CP hydrogel blend could serves as a potential therapeutic constituent towards cartilage regeneration. In addition, delivery of ADMSCs through this hydrogels could be a better choice for accelerated healing of cartilage injury.

The vial-inversion test and rheological study indicate gelation of CP hydrogel system at 32.2 °C. The system shows Newtonian fluid-like consistency below 32 °C, which turns in to gel with radical increase in storage modulus above critical gelation temperature^31^. The point at temperature 32.2 °C may be defined as the critical gelation temperature (*T*_gel_). *T*_gel_ is normally defined as a temperature where yield stress appears due to transition of a Newtonian fluid to non-Newtonian fluid by developing a 3D spatial “jammed microstructure”^32^. The viscosity sharply increased as a function of temperature during the growth of micelles with the conversion of the homogeneous fluid into a biphasic system^33^. Finally, the viscosity is platitude off and the gelation process reached equilibrium^34^. Further, pluronic F127 hydrogel with a concentration of 20% w/v could also be explored for bio-printing application owing to its rapid gelation under physiological condition^35^.

For assessing cytocompatibility of CP hydrogel and its capacity to induce differentiation of mesenchymal stem cells towards chondrogenic lineage, Capra adipose tissue-derived ADMSCs were employed in the study. The ADMSCs were isolated and characterized according to standards of the International Federation for Adipose Therapeutics and Science (IFATS) and International Society for Cellular Therapy (ISCT)^36^. Cells were identified by expression of positive cell surface markers (CD44, CD 90, CD 73, and CD 105) and absence of negative cell surface markers (CD 31 and CD 45), respectively. The later markers are typically expressed in hematopoietic stem cells. The expression of positive surface markers confirmed that the isolated cells were MSCs. In addition to this, trilineage differentiation potential of isolated ADMSCs was further characterized by the accumulation of oil droplets in cells cytoplasm during adipogenic differentiation study, formation of Alcian blue-positive micro-tissues during chondrogenic differentiation study and deposition of the mineralized matrix during differentiation of osteogenic lineage. The trilineage differentiation potential of ADMSCs was further confirmed by qualitative fluorescence staining as well as RT-PCR studies^22^.

After five days of ADMSCs culture, the formulated hydrogel was found to be nontoxic, while exhibited good cell adhesion potential and rate of proliferation without extensive alteration of cell morphology as assessed through Rhodamine-DAPI and Live-Dead staining.

The effects of CP hydrogel towards regeneration of damaged cartilage was further analyzed by culturing ADMSCs in the 3D environment of the hydrogel^37^. Upregulation of COLII related genes in RT-PCR studies as well as higher accumulation sGAG using CP hydrogel as compared to that of F127 hydrogel clearly indicate that CP hydrogel has a comparatively higher capacity to induce differentiation of ADMSCs towards chondrogenic lineage along with cartilage micro-tissue formation in 3D environment^34^. It may also be assumed that Pluronic F-127 has a considerable role to provide a 3D environment for differentiation of ADMSCs to cartilage lineage^37^. Herein, the blended CP hydrogel has an encouraging result to be used as injectable hydrogel for cartilage regeneration and also to perform as a stem cell delivery vehicle by minimal invasion^38^.

The study includes isolation of COLII from bio-waste material, characterization, and identification by a novel approach and its application as injectable hydrogel towards cartilage regeneration through a facile technique. The optimized process is a simple and cost-effective one for faster isolation of COLII thereby offering an economical therapeutic option. Additionally, a newly developed mass spectrometric analytic method was used to detect the position and expressional alteration of post translationally modified Hyp in Gly–X–Y motif as a signature for plausible association with arthritis and could be an early marker to identify the onset of cartilage disorder. It might also be assumed that the mapping of Hyp position in signature triplet would identify the COLII derived from other sources. The application of COLII in hydrogel form could be a promising stem cell delivery vehicle as well as an alternative therapeutic option towards accelerating cartilage regeneration.

## Materials and methods

### Collection and processing of *Capra* ears

*Capra* ears were freshly collected from a local slaughter house and washed with water. *Capra* ear skin was removed considerably through treatments with hypertonic (1 M) NaCl solution prepared in 0.05 M Tris-HCl, pH 7.5, followed by 0.04% pepsin digestion at 37 °C for 72 h and washed with PBS thrice.

### Isolation of COLII from *Capra* ear

The skin free *Capra* ear cartilage was treated with NaOH (0.1 M) for 2–3 h followed by mechanical grinding, digested with acetic acid/pepsin solution, salt precipitation using 1.2 M NaCl and centrifuged at 8000 rpm for pelletization (Supplementary Fig. 1). The protein pellet was collected and purified using dialysis membrane (12–14 kDa molecular cut-off) with repeated changes of deionized water. The final product was lyophilized and stored at −20 °C until further use^16^.

### Yield calculation of collagen II

Yield of the acid/pepsin-solubilized isolated proteins under different digestion conditions was calculated as per equation 1. Percent Hyp was determined according to the method reported elsewhere^10^.

1 $$\% {\mathrm{Yield}}\,({\mathrm{dry}}\,{\mathrm{weight}}\,{\mathrm{basis}}) = \frac{{{\mathrm{Dry}}\,{\mathrm{weight}}\,{\mathrm{collagen}} \times 100}}{{({\mathrm{Wet}}\,{\mathrm{weight}}\,{\mathrm{of}}\,{\mathrm{sample}} - {\mathrm{Moisture}}\,{\mathrm{content}}\,{\mathrm{of}}\,{\mathrm{sample}})}}$$

### SDS-PAGE and western nlot analysis

SDS–PAGE was performed using 4% (w/v) stacking gel and 8 % (w/v) separating gel. The purified protein was mixed with 6× loading dye at 1:3 (w/v) ratio and subsequently loaded in stacking gel. Protein molecular marker (MW range 25–250 kDa; BioRad, India) were run in a separate well along with samples. Electrophoresis was performed using mini dual vertical electrophoresis unit at for 90 V for 2 h (BioRad, India). The obtained band was observed by Coomassie blue staining^17,39^.

After separation of proteins through SDS-PAGE, the gel was electro-blotted onto a nitrocellulose membrane (Millipore, USA) in tris-glycine buffer at 90 V for 2 h. Membranes were incubated with anti-collagen II antibody and anti-elastin antibody (1:5000) (Abcam, USA) at 4 °C over-night after blocking with 3% BSA in PBS. The blots were washed with PBST (0.05% Tween 20 in PBS) and incubated with horseradish peroxidase (HRP) conjugated secondary antibody (1:6000) for 2 h at RT. BioRad ECL-western blotting substrate was used to visualize the immune-reactive proteins as per the manufacturer’s instructions (Thermo Scientific, USA). Images were observed by chemo-luminescence.

Fourier transform infrared spectroscopy (FTIR): FTIR spectrum of purified protein was collected through KBr pelletization technique in the range of 4000–700 cm^−1^ using Thermo Nicolet Spectrophotometer (Model NEXUS-870; Thermo Nicolet Corporation, Madison, WI)

Circular dichroism (CD): CD spectra were collected to analyze the secondary structure of acid/pepsin-solubilized protein fraction under different digestion conditions. COLII (1 mg) was dissolved in 1:10 (w/v) acetic acid (0.05 M) solution and placed into a quartz cell with a path length of 1 mm. CD spectra of protein solution were recorded from 280 to 190 nm with a step size of 1.0 nm and bandwidth 1.0 nm at scan speed 100 nm/min after subtraction of solvent spectrum.

Scanning electron microscopy (SEM): FESEM (EVO 60, Carl Zeiss, Germany) of the purified protein was carried out after gold coating using plasma coater for the 30 s under high vacuum.

Amino acid profiling: For amino acid analysis, purified protein was hydrolyzed with 6 N HCl for 24 h at 120 °C. The resultant mixture was analysed by an Agilent 1260 HPLC system (Agilent, USA) with a fluorescent detector (FLD) after derivatisation with OPA (O-phthalaldehyde) (SRL, India). For proline and hydroxyproline identification, 9-fluorenylmethoxycarbonyl (FMOC-Cl) (SRL, India) was applied to derivatise the sample^40^. The derivatized sample was loaded (50 µl) onto an HPLC column (ZORBAX-SB-C-18 column (250 × 4.6 m, 5 micron particle size) using sample injector (Agilent 1260). For gradient elution method, the column was eluted using 0.01 M Na_2_HPO_4_ buffer and acetonitrile (100%) as a mobile phase solvent system (Supplementary Table 4). The flow rate was maintained at 1 ml/min. Data from the system was collected and evaluated using Agilent open LAB control panel software. Amino acid from sample and standard was quantified via comparison to the retention time and absorbance. The amino acid content was expressed as the number of residues/1000 residues.

Processing of protein bands for in-gel digestion: After SDS-PAGE electrophoresis, the required protein band was excised (1–2 mm) and digested with sequence graded trypsin according to the protocol published elswhere^41^. Briefly, the excised band was washed with distilled water (D/W) followed by destaing (100 mM ammonium bicarbonate in 100% acetonitrile at 1:1 v/v) for 30 m at 37 °C. The shrunk gel pieces were washed with acetonitrile followed by air-dry. For reduction and alkylation of protein, 20 µl of 10 mM DTT was added into gel pieces for 45 min at 56 °C followed by storing at RT and subsequently 20 µl of 55 mM iodoacetamide (IAA) was added into the gel pieces for 30–60 m in dark place. Excess IAA was removed by shrinking the gel pieces and subsequent washing with acetonitrile. The dehydrated gel pieces were digested with sequence graded trypsin at 37 °C air bath for overnight (Product number V5111, Promega gold, Madison, WI) to cleave the protein at specific peptide bonds depending on the amino-acid sequences^9^. Trypsin powder (100 µg/vial) was resuspended according to the manufacture’s protocol (Promega gold, Madison, WI). To stop the trypsin reaction, peptide extraction was performed by addition of 50 µl of extraction solution (60% acetonitrile, 0.1% TFA) to gel pieces thrice followed by centrifugation at 12,000 × *g* for 20 s. Supernant was collected and lyophilized for further use. Lyophilized peptides were mixed with equal vol. of matrix solution of α-cyano-4-hydroxycinnamic acid (Bruker Daltonics, Germany) in 30% acetonitrile and 70% TFA (0.1% TFA in MQ water) and spotted on to the Anchor chip target plate (600/384F, Bruker Daltonics). The spot was allowed to dry for 30 min before MALDI-MS analysis.

MALDI-TOF/TOF-MS analysis: The dried spots were analyzed for identification of COLII by using Ultraflextreme mass spectrometer (Bruker Daltonics, Germany) for MALDI MS and MS/MS analysis. Different area of sample spot on MALDI target plate was selected for data acquisition.

Mass spectrometry data analysis: Data acquisition was performed on reflector mode and the spectra were processed using Flex analysis 3.2 software (Bruker Daltonics, Germany) followed by MS/MS ion search using MASCOT server (Matrix Science)^42^. The search parameters for MALDI-TOF/TOF MS analysis were set to be 2 Da for fragment ion tolerance and 100 ppm for peptide mass tolerance.

Tandem mass spectrometry: Selected peptides were further fragmented using collision induced dissociation (CID) method. The resultant MS/MS spectra were annotated and searched against the SwissProt database using MASCOT search engine. Peptide sequences were determined by interpreting the data resulting from fragment ion analysis^39,43,44^ and proline oxidation (addition of 16 Da to the mass of proline residue) was considered as a modified parameter in MASCOT search engine for detection of Hyp PTM present in COLIIA1 peptide sequences.

Preparation of CP hydrogel: An appropriate amount (20%) of Pluronic F127 copolymer and collagen (3%) weight percentage was dissolved in DMEM media by cold method. In brief, prior to preparing the CP hydrogel, the aforementioned components were initially preserved at 4 °C to achieve a homogeneous pre-gel mix. The pre-gel mix was shaken vigorously and kept back at 4 °C for overnight. Being a thermo-responsive block copolymer, F127 follows sol–gel transition and obeys the rule of mixture over an alternating range of temperature. After completion dissolution of the polymer, the system was allowed to stand at 4 °C for 24 h for degassing and form a clear translucent dispersion for further application.

Isolation and identification of ADMSCs from Capra hircus: Necessary approval for the isolation of ADMSCs was obtained from the Institutional Committee for Stem cell Research and Therapy (ICSRT) of Indian Institute of Engineering Science and Technology, Shibpur, India (IIEST). *Capra hircus* adipose tissue-derived ADMSCs were cultured as per the protocol reported elsewhere^45^. Briefly, fresh adipose tissues of *Capra hircus* were collected from butcher’s shop. The collected tissue was washed with 70% alcohol for 10–20 s, followed by washing (4×) with antibiotic-PBS solution. The tissue was then chopped and vortexes with an equal volume of PBS for three times at one minute’s interval. The viscous solution was centrifuged at 900 × *g* and pellet containing small vascular fraction (SVF) was collected and washed with PBS. Cells were transferred to T25 flask containing DMEM media (Gibco, USA), 10% FBS (Invitrogen, USA) and 1% antibiotic-antimitotic solution (Invitrogen, USA). The cells were cultured in CO_2_ incubator until confluence reached 70–80%.

The isolated cells were identified by immunofluorescence staining using ADMSCs specific rabbit polyclonal CD 44 and CD 31 antibodies (1:300; Abcam) and Alexa Fluor 488 labeled goat anti-rabbit secondary antibody (1:300; Life Technologies) along with DAPI counter-staining as per the protocol reported elsewhere^46^. For RT-PCR study, RNA was harvested from the isolated ADMSCs using RNA extraction kit (Invitrogen, India) according to the manufacturer’s instruction. Synthesis of cDNA and PCR amplification were carried out as per the manufacturer’s protocol (Thermo Scientific, USA) using primers as enlisted in Supplementary Table 5. Gene expression analysis was performed according to our previously reported protocol^46^ and PCR products were analysed by 1% agarose gel-electrophoresis.

Assessment of trilineage differential potential of ADMSCs: For adipogenic differentiation, ADMSCs were plated at a density of 1 × 10^5^ in each well of 6-well plate. After adhesion of cells, the DMEM media was replaced with lineage-specific differentiation medium. For adipogenic differentiation media contained: DMEM (high glucose), 10 ml FBS, 0.0393 mg dexamethasone (Himedia), 11.1 mg 3-isobutyl-1-methylxanthine (Sigma), 7.16 mg indomethacin (Sigma) and 5.73 mg insulin (Sigma)^22^. Chondrogenic differentiation media consisted: DMEM (high glucose), 2–5% FBS (Gibco, USA), 0.00393 mg dexamethasone (Himedia, India), 5.79 mg ascorbic acid-2-phosphate (Sigma, USA), 1 ml ITS-X (Life Technologies), 0.402 mg l-proline and 10 µl TGF-β3 (Gibco, USA)^45^. Osteogenic differentiation medium composed of DMEM (high glucose), 10% FBS, 0.00393 mg dexamethasone (Himedia, India), 216 mg β-glycerophosphate (Sigma, USA) and 5 mg ascorbic acid (Sigma, USA)^47^.

Media was replaced in every 3 days up to 21 days of differentiation study. At the end of the experiment, cells were rinsed with PBS and fixed with 4% paraformaldehyde for 60 min followed by washing with distilled water (D/W). After adipogenic differentiation, fixed cells were rinsed with 60% isopropanol followed by incubation with Oil Red O solution for 5 min. The cells were washed with D/W followed by counterstaining with Hematoxylin for 10–20 s. The fixed cells after chondrogenic differentiation were stained with 1% Alcian Blue solution prepared in 3% acetic acid (pH 2.5) for 30 min followed by counterstaining with nuclear fast red for 5 min. The cells underwent osteogenic induction were stained with 2% Alizarin Red S solution at room temperature for 10 min followed by washing with D/W. After staining, cells were visualized under the light microscope (AxioVision, Zeiss, Germany)^22,45^.

In vitro cytocompatibility study: CP hydrogel was formed within the wells of a 24-well cell culture plate surface (1 ml/well) for in situ gelations at 37 °C for 5 min. The harvested ADMSCs from passage three were seeded in equal numbers (50,000 cells/well) on CP hydrogel as well as tissue culture plate (TCP) as a control. The cells were cultured in DMEM (Himedia, India) media containing 10% FBS and 1% antibiotic solution at 37 °C in 5% CO_2_ atmosphere (Heracell 150i, Thermo, USA).

Cytotoxicity and cell proliferation assay were evaluated by Rhodamin-DAPI and Live-dead assay, as previously described^31^. The assay of the cells on the samples and control were examined after 1, 3, and 5 days studies. After 1, 3, and 5 days, cell morphology was evaluated by rhodamine-phalloidin (Life Technologies, Invitrogen) and DAPI (4′,6-diamidino-2-phenylindole, (Life Technologies, Invitrogen) staining as of manufacturer’s statement. Briefly, cells were fixed in 4% paraformaldehyde, permeabilized using Triton-X100 followed by blocking the nonspecific sites using 1% BSA (Sigma). For cytoskeleton staining, rhodamine–phalloidin dye solution was added to the samples and incubated for 30 min followed by washing thrice with PBS. DAPI (nucleus staining) was also applied for 5 min and rinsed with PBS followed by imaging under the fluorescent microscope (Carl Zeiss, Germany) using ZEN software. The live-dead assay was performed by using Live–Dead staining kit (Invitrogen, USA). The cell-gel construct was incubated for 30 min at room temperature in a solution containing 2 µM calcein aceto-methoxy (AM) and 4 µM ethidium homodimer. After incubation, the cell-gel assembly was observed using fluorescent microscope (Carl Zeiss, Germany) with excitation filters of 450–490 nm (green, Calcein AM) and 510–560 nm (red, ETD-1) using ZEN software.

Three-dimensional cell culture for chondrogenic differentiation: The confluent ADMSCs were trypsinized, centrifuged, and suspended in fresh medium and counted in a haemocytometer. Cells were again centrifuged and cell pellets were re-suspended in sterile CP hydrogel and TCP was used as controls. The hydrogel solution was kept on below gelation temperature to prevent premature gelation during cell-gel construction formation. The differentiation study was performed in standard 6-well plate with the addition of chondrogenic differentiation media. The cell-hydrogel construct and TCP was cultured at 37 °C, 5% CO_2_ incubator and media were replaced every 3rd day. The differentiation was assessed after 21 days cultured by RT-PCR. RNA isolation, cDNA synthesis and COLII specific gene amplification were performed by above mentioned RT-PCR protocol.

sGAG assay by Alcian blue quantitative method: After chondrogenic differentiation of ADMSCs within CP hydrogel as well as TCP as control, the quantitative sGAG analysis was carried out by Alcian blue method according to the protocol reported elsewhere^48,49^. Briefly, the samples were digested with 0.1 M phosphate buffer solution (pH 6.8) containing 10 mM cysteine hydrochloride (Sigma, USA), 125 µg/ml papain (Sigma, USA) and 2 mM Na_2_EDTA (Sigma, USA) at 60 °C for 12 h. After digestion, the solution was centrifuged at 15,000 × *g* for 20 min and the supernatant was collected for further analysis. Alcian Blue 8GS (SRL, India) dye stock solution (1 g Alcian Blue 8GS dye in 100 ml of 18 mM H_2_SO_4_) was prepared in which 10% dye stock solution including 0.25% Triton X-100, 0.018 M H_2_SO_4_ was used to make a working solution. Guanidine solution (4 M and 8 M) was prepared with 0.027 M H_2_SO_4_ and 0.375% Triton X-100. Standard curve was made using different concentrations (100, 200, 400, 600, 800 µg/ml) of chondroitin sulfate. Alcian blue solution was mixed with sample and standard followed by vortexes for 5 m and centrifuged at 16,000 × *g* for 10 m at 4 °C. The obtained pellet was dissolved in 500 µl of 8 M guanidine HCl by vigorous vortexing followed by centrifugation at 16,000 × *g* for 3 m. The absorbance of the Alcian blue-sGAGs complex was measured at 595 nm wavelength using iMarkt microplate reader and sGAGs contents were estimated from the standard curve.

### Statistical analysis of the data

The experiments were carried out in triplicate. The data were expressed as the means ± standard error of mean and were statistically tested by performing *t*-tests using SPSS 19.0 software (Chicago, IL, USA). *P* < 0.001 was considered statistically significant.

### Reporting summary

Further information on experimental design is available in the Nature Research Reporting Summary linked to this article.

## Supplementary information

Supplementary Information

Reporting Summary

## Data Availability

The mass spectrometry proteomics data have been deposited to the ProteomeXchange Consortium via the PRIDE^49^ partner repository with the dataset identifier PXD012928 and PXD012911. Other data generated in this study is available from the corresponding author upon reasonable request.
