# Supplementary material for: Isolation and mass spectrometry based hydroxyproline mapping of type II collagen derived from Capra hircus ear cartilage
Source: Commun Biol. 2019 Apr 29;2:146. doi: 10.1038/s42003-019-0394-6 (PMC6488623; doi:10.1038/s42003-019-0394-6)
Supplement: Supplementary file 1 — Supplementary Information [file 42003_2019_394_MOESM1_ESM.pdf]

Supplementary

Isolation and mass spectrometry based hydroxyproline mapping of type II collagen derived from *Capra hircus* ear cartilage

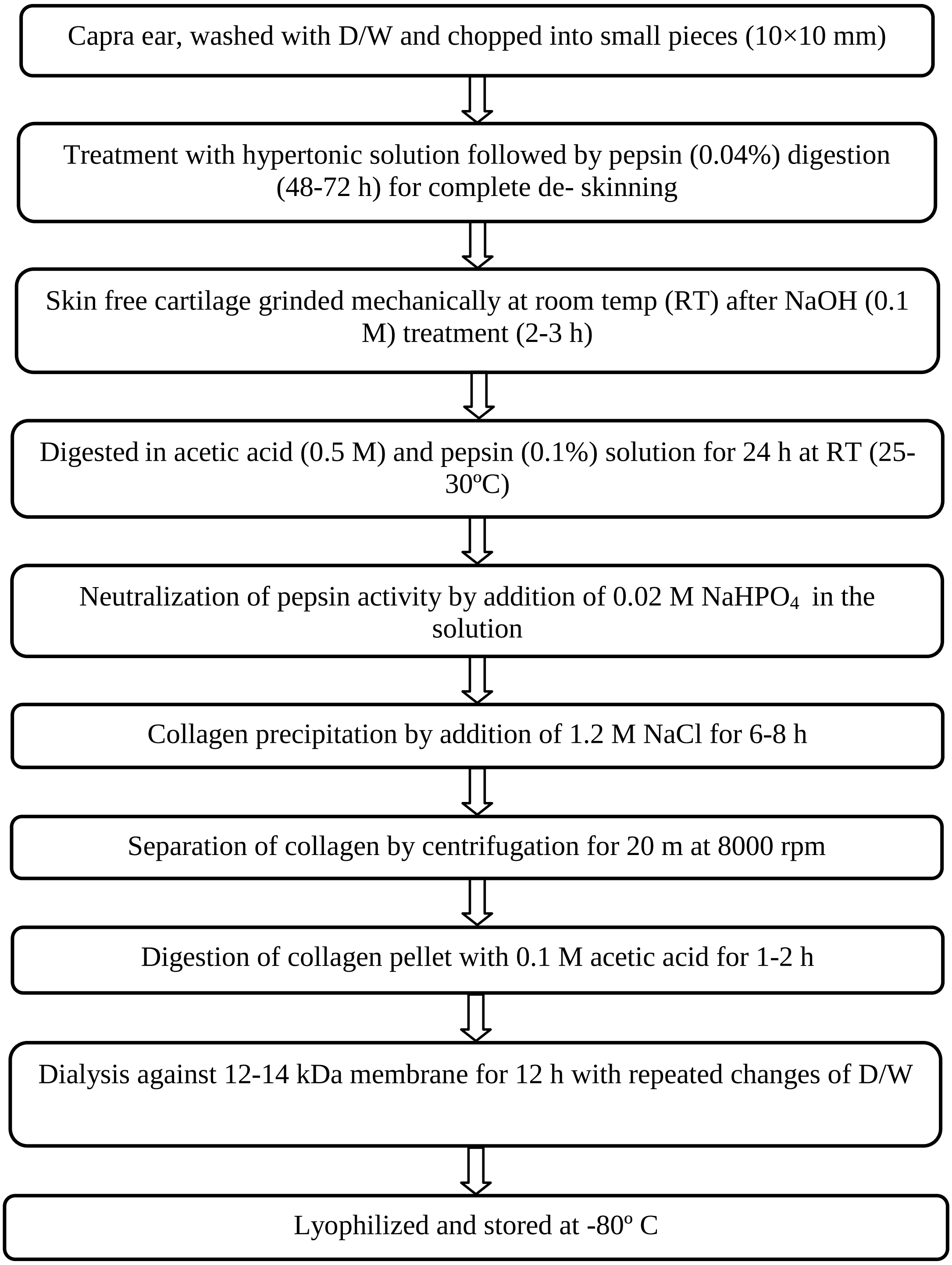

Supplementary Figure 1. Schematic representation of collagen II isolation from Capra ear.

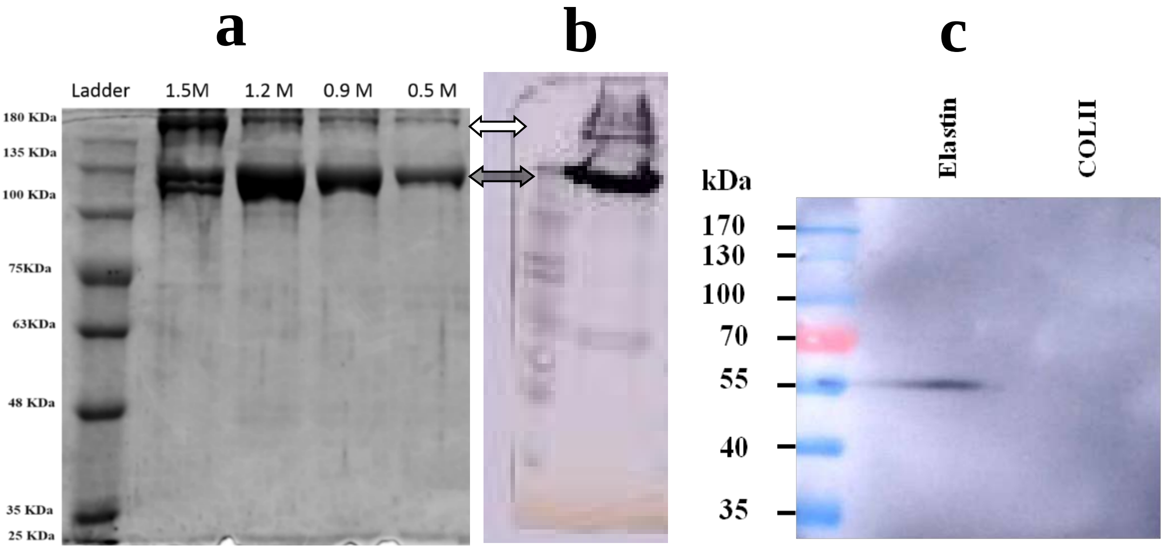

Supplementary Figure 2. Identification of purified COLII by SDS-PAGE and western blot analysis. (a) SDS-PAGE showing the isolated COLII with two prominent bands where filled arrow indicating α chain and open arrow indicating β chain, (b) α chain was identified using anti-COLII antibody using western blot analysis and (c) showing prominent band of bovine elastin (Sigma, USA) as a control and absence of band in *Capra* ear derived COLII indicating isolated protein was devoid of elastin.

```
Capra_COLIIA1    MIRLGAPQTLVLLTLLVAAVLRCHGQDVQKAGSCVQDGQRYNDKDVWKPEPCRICVCDTG 60
Bos_COLIIA1      MIRLGAPQTLVLLTLLVAAVLRCHGQDVQKAGSCVQDGQRYNDKDVWKPEPCRICVCDTG 60
Homo_COLIIA1     MIRLGAPQTLVLLTLLVAAVLRCQGQDVQEAGSCVQDGQRYNDKDVWKPEPCRICVCDTG 60
                 ***********************:*****:******************************

Capra_COLIIA1    TVLCDDIICEDMKDCLSPETPFGECCPICSADLPTASGQPGPKGQKGEPGDIKDIVGPKG 120
Bos_COLIIA1      TVLCDDIICEDMKDCLSPETPFGECCPICSADLPTASGQPGPKGQKGEPGDIKDIVGPKG 120
Homo_COLIIA1     TVLCDDIICEDVKDCLSPEIPFGECCPICPTDLATASGQPGPKGQKGEPGDIKDIVGPKG 120
                 ***********:******* ********* :** **************************

Capra_COLIIA1    PPGPQGPAGEQGPRGDRGDKGEKGAPGPRGRDGEPGTPGNPGPPGPPGPPGPPGLGGNFA 180
Bos_COLIIA1      PPGPQGPAGEQGPRGDRGDKGEKGAPGPRGRDGEPGTPGNPGPPGPPGPPGPPGLGGNFA 180
Homo_COLIIA1     PPGPQGPAGEQGPRGDRGDKGEKGAPGPRGRDGEPGTPGNPGPPGPPGPPGPPGLGGNFA 180
                 ************************************************************

Capra_COLIIA1    AQMAGGFDEKAGGAQMGVMQGPMGPMGPRGPPGPAGAPGPQGFQGNPGEPGEPGVSGPMG 240
Bos_COLIIA1      AQMAGGFDEKAGGAQMGVMQGPMGPMGPRGPPGPAGAPGPQGFQGNPGEPGEPGVSGPMG 240
Homo_COLIIA1     AQMAGGFDEKAGGAQLGVMQGPMGPMGPRGPPGPAGAPGPQGFQGNPGEPGEPGVSGPMG 240
                 ***************:********************************************

Capra_COLIIA1    PRGPPGPPGKPGDDGEAGKPGKSGERGPPGPQGARGFPGTPGLPGVKGHRGYPGLDGAKG 300
Bos_COLIIA1      PRGPPGPPGKPGDDGEAGKPGKSGERGPPGPQGARGFPGTPGLPGVKGHRGYPGLDGAKG 300
Homo_COLIIA1     PRGPPGPPGKPGDDGEAGKPGKAGERGPPGPQGARGFPGTPGLPGVKGHRGYPGLDGAKG 300
                 **********************:*************************************

Capra_COLIIA1    EAGAPGVKGESGSPGENGSPGPMGPRGLPGERGRTGPAGAAGARGNDGQPGPAGPPGPVG 360
Bos_COLIIA1      EAGAPGVKGESGSPGENGSPGPMGPRGLPGERGRTGPAGAAGARGNDGQPGPAGPPGPVG 360
Homo_COLIIA1     EAGAPGVKGESGSPGENGSPGPMGPRGLPGERGRTGPAGAAGARGNDGQPGPAGPPGPVG 360
                 ************************************************************

Capra_COLIIA1    PAGGPGFPGAPGAKGEAGPTGARGPEGAQGPRGEPGTPGSPGPAGAAGNPGTDGIPGAKG 420
Bos_COLIIA1      PAGGPGFPGAPGAKGEAGPTGARGPEGAQGPRGEPGTPGSPGPAGAAGNPGTDGIPGAKG 420
Homo_COLIIA1     PAGGPGFPGAPGAKGEAGPTGARGPEGAQGPRGEPGTPGSPGPAGASGNPGTDGIPGAKG 420
                 **********************************************:*************

Capra_COLIIA1    APGPAGEEGKRGARGEPGGAGPAGPPGERGAPGNRGFPGQDGLAGPKGAPGERGPSGLAG 540
Bos_COLIIA1      APGPAGEEGKRGARGEPGGAGPAGPPGERGAPGNRGFPGQDGLAGPKGAPGERGPSGLAG 540
Homo_COLIIA1     APGPAGEEGKRGARGEPGGVGPIGPPGERGAPGNRGFPGQDGLAGPKGAPGERGPSGLAG 540
                 *******************.** *************************************

Capra_COLIIA1    PKGANGDPGRPGEPGLPGARGLTGRPGDAGPQGKVGPSGAPGEDGRPGPPGPQGARGQPG 600
Bos_COLIIA1      PKGANGDPGRPGEPGLPGARGLTGRPGDAGPQGKVGPSGAPGEDGRPGPPGPQGARGQPG 600
Homo_COLIIA1     PKGANGDPGRPGEPGLPGARGLTGRPGDAGPQGKVGPSGAPGEDGRPGPPGPQGARGQPG 600
                 ************************************************************

Capra_COLIIA1    VMGFPGPKGANGEPGKAGEKGLPGAPGLRGLPGKDGETGAAGPPGPAGPAGERGEQGAPG 660
Bos_COLIIA1      VMGFPGPKGANGEPGKAGEKGLPGAPGLRGLPGKDGETGAAGPPGPAGPAGERGEQGAPG 660
Homo_COLIIA1     VMGFPGPKGANGEPGKAGEKGLPGAPGLRGLPGKDGETGAAGPPGPAGPAGERGEQGAPG 660
                 ************************************************************

Capra_COLIIA1    PSGFQGLPGPPGPPGEGGKPGDQGVPGEAGAPGLVGPRGERGFPGERGSPGSQGLQGARG 720
Bos_COLIIA1      PSGFQGLPGPPGPPGEGGKPGDQGVPGEAGAPGLVGPRGERGFPGERGSPGSQGLQGARG 720
Homo_COLIIA1     PSGFQGLPGPPGPPGEGGKPGDQGVPGEAGAPGLVGPRGERGFPGERGSPGAQGLQGPRG 720
                 ***************************************************:***** **

Capra_COLIIA1    LPGTPGTDGPKGAAGPAGPPGAQGPPGLQGMPGERGAAGIAGPKGDRGDVGEKGPEGAPG 780
Bos_COLIIA1      LPGTPGTDGPKGAAGPAGPPGAQGPPGLQGMPGERGAAGIAGPKGDRGDVGEKGPEGAPG 780
Homo_COLIIA1     LPGTPGTDGPKGASGPAGPPGAQGPPGLQGMPGERGAAGIAGPKGDRGDVGEKGPEGAPG 780
                 *************:**********************************************

Capra_COLIIA1    KDGGRGLTGPIGPPGPAGANGEKGEVGPPGPAGTAGARGAPGERGETGPPGPAGFAGPPG 840
Bos_COLIIA1      KDGGRGLTGPIGPPGPAGANGEKGEVGPPGPAGTAGARGAPGERGETGPPGPAGFAGPPG 840
Homo_COLIIA1     KDGGRGLTGPIGPPGPAGANGEKGEVGPPGPAGSAGARGAPGERGETGPPGPAGFAGPPG 840
                 *********************************:**************************

Capra_COLIIA1    ADGQPGAKGEQGEAGQKGDAGAPGPQGPSGAPGPQGPTGVTGPKGARGAQGPPGATGFPG 900
Bos_COLIIA1      ADGQPGAKGEQGEAGQKGDAGAPGPQGPSGAPGPQGPTGVTGPKGARGAQGPPGATGFPG 900
Homo_COLIIA1     ADGQPGAKGEQGEAGQKGDAGAPGPQGPSGAPGPQGPTGVTGPKGARGAQGPPGATGFPG 900
                 ************************************************************

Capra_COLIIA1    AAGRVGPPGSNGNPGPPGPPGPSGKDGPKGARGDSGPPGRAGDPGLQGPAGPPGEKGEPG 960
Bos_COLIIA1      AAGRVGPPGSNGNPGPPGPPGPSGKDGPKGARGDSGPPGRAGDPGLQGPAGPPGEKGEPG 960
Homo_COLIIA1     AAGRVGPPGSNGNPGPPGPPGPSGKDGPKGARGDSGPPGRAGEPGLQGPAGPPGEKGEPG 960
                 ******************************************:*****************

Capra_COLIIA1    DDGPSGPDGPPGPQGLAGQRGIVGLPGQRGERGFPGLPGPSGEPGKQGAPGASGDRGPPG 1020
Bos_COLIIA1      DDGPSGPDGPPGPQGLAGQRGIVGLPGQRGERGFPGLPGPSGEPGKQGAPGASGDRGPPG 1020
Homo_COLIIA1     DDGPSGAEGPPGPQGLAGQRGIVGLPGQRGERGFPGLPGPSGEPGKQGAPGASGDRGPPG 1020
                 ****** :****************************************************

Capra_COLIIA1    PVGPPGLTGPAGEPGREGSPGADGPPGRDGAAGVKGDRGETGAVGAPGAPGPPGSPGPAG 1080
Bos_COLIIA1      PVGPPGLTGPAGEPGREGSPGADGPPGRDGAAGVKGDRGETGAVGAPGAPGPPGSPGPAG 1080
Homo_COLIIA1     PVGPPGLTGPAGEPGREGSPGADGPPGRDGAAGVKGDRGETGAVGAPGAPGPPGSPGPAG 1080
                 ************************************************************

Capra_COLIIA1    PVGKQGDRGEAGAQGPMGPAGPAGARGMPGPQGPRGDKGETGEAGERGLKGHRGFTGLQG 1140
Bos_COLIIA1      PIGKQGDRGEAGAQGPMGPAGPAGARGMPGPQGPRGDKGETGEAGERGLKGHRGFTGLQG 1140
Homo_COLIIA1     PTGKQGDRGEAGAQGPMGPSGPAGARGIQGPQGPRGDKGEAGEPGERGLKGHRGFTGLQG 1140
                 * *****************:*******: ***********:** ****************

Capra_COLIIA1    LPGPPGPSGDQGASGPAGPSGPRGPPGPVGPSGKDGANGIPGPIGPPGPRGRSGETGPAG 1200
Bos_COLIIA1      LPGPPGPSGDQGASGPAGPSGPRGPPGPVGPSGKDGANGIPGPIGPPGPRGRSGETGPAG 1200
Homo_COLIIA1     LPGPPGPSGDQGASGPAGPSGPRGPPGPVGPSGKDGANGIPGPIGPPGPRGRSGETGPAG 1200
                 ************************************************************

Capra_COLIIA1    PPGNPGPPGPPGPPGPGIDMSAFAGLGQREKGPDPLQYMRADEAAGNLRQHDAEVDATLK 1260
Bos_COLIIA1      PPGNPGPPGPPGPPGPGIDMSAFAGLGQREKGPDPLQYMRADEAAGNLRQHDAEVDATLK 1260
Homo_COLIIA1     PPGNPGPPGPPGPPGPGIDMSAFAGLGPREKGPDPLQYMRADQAAGGLRQHDAEVDATLK 1260
                 *************************** **************:***.*************

Capra_COLIIA1    SLNNQIESLRSPEGSRKNPARTCRDLKLCHPEWKSGDYWIDPNQGCTLDAMKVFCNMETG 1320
Bos_COLIIA1      SLNNQIESLRSPEGSRKNPARTCRDLKLCHPEWKSGDYWIDPNQGCTLDAMKVFCNMETG 1320
Homo_COLIIA1     SLNNQIESIRSPEGSRKNPARTCRDLKLCHPEWKSGDYWIDPNQGCTLDAMKVFCNMETG 1320
                 ********:***************************************************

Capra_COLIIA1    ETCVYPNPASVPKKNWWSSKSKDKKHIWFGETINGGFHFSYGDDNLAPNTANVQMTFLRL 1380
Bos_COLIIA1      ETCVYPNPASVPKKNWWSSKSKDKKHIWFGETINGGFHFSYGDDNLAPNTANVQMTFLRL 1380
Homo_COLIIA1     ETCVYPNPANVPKKNWWSSKSKEKKHIWFGETINGGFHFSYGDDNLAPNTANVQMTFLRL 1380
                 *********.************:*************************************

Capra_COLIIA1    LSTEGSQNITYHCKNSIAYLDEAAGNLKKALLIQGSNDVEIRAEGNSRFTYTVLKDGCTK 1440
Bos_COLIIA1      LSTEGSQNITYHCKNSIAYLDEAAGNLKKALLIQGSNDVEIRAEGNSRFTYTVLKDGCTK 1440
Homo_COLIIA1     LSTEGSQNITYHCKNSIAYLDEAAGNLKKALLIQGSNDVEIRAEGNSRFTYTALKDGCTK 1440
                 ****************************************************.*******

Capra_COLIIA1    HTGKWGETVIEYRSQKTSRLPIIDIAPMDIGGPEQEFGVDIGPVCFL 1487
Bos_COLIIA1      HTGKWGKTMIEYRSQKTSRLPIIDIAPMDIGGPEQEFGVDIGPVCFL 1487
Homo_COLIIA1     HTGKWGKTVIEYRSQKTSRLPIIDIAPMDIGGPEQEFGVDIGPVCFL 1487
                 ******:*:**************************************
```

Supplementary Figure 3. Amino acid sequence alignment of COLIIA1 from *Capra hircus, Bos taurus* and *Homo sapiens* showing 98% identity.

1 MIRLGAPQTL VLLTLLVAAV LRCHGQDVQK AGSCVQDGQR YNDKDVWKPE

51 PCRICVCDTG TVLCDDIICE DMKDCLSPET PFGECCPICS ADLPTASGQP

101 GPKGQKGEPG DIKDIVGPKG PPGPQGPAGE QGPRGDRGDK **GEKGAPGPR**G

151 RDGEPGTPGN PGPPGPPGPP GPPGLGGNFA AQMAGGFDEK **AGGAQMGVMQ**

201 **GPMGPMGPR**G PPGPAGAPGP QGFQGNPGEP GEPGVSGPMG PRGPPGPPGK

251 PGDDGEAGKP GKSGER**GPPG** **PQGARGFPGT** **PGLPGVK**GHR GYPGLDGAKG

301 EAGAPGVKGE SGSPGENGSP GPMGPRGLPG ERGR**TGPAGA** **AGAR**GNDGQP

351 GPAGPPGPVG PAGGPGFPGA PGAKGEAGPT GARGPEGAQG PRGEPGTPGS

401 PGPAGAAGNP GTDGIPGAK**G** **SAGAPGIAGA** **PGFPGPR**GPP GPQGATGPLG

451 PKGQTGEPGI AGFKGEQGPK GEPGPAGPQG APGPAGEEGK RGAR**GEPGGA**

501 **GPAGPPGERG** **APGNR**GFPGQ DGLAGPK**GAP** **GERGPSGLAG** **PKGANGDPGR**

551 **PGEPGLPGAR** **GLTGRPGDAG** **PQGKVGPSGA** **PGEDGRPGPP** **GPQGAR**GQPG

601 VMGFPGPKGA NGEPGKAGEK **GLPGAPGLRG** **LPGKDGETGA** **AGPPGPAGPA**

651 **GER**GEQGAPG PSGFQGLPGP PGPPGEGGKP GDQGVPGEAG APGLVGPRGE

701 R**GFPGER**GSP GSQGLQGAR**G** **LPGTPGTDGP** **KGAAGPAGPP** **GAQGPPGLQG**

751 **MPGERGAAGI** **AGPK**GDRGDV GEKGPEGAPG KDGGRGLTGP IGPPGPAGAN

801 GEKGEVGPPG PAGTAGARGA PGER**GETGPP** **GPAGFAGPPG** **ADGQPGAK**GE

851 QGEAGQKGDA GAPGPQGPSG APGPQGPTGV TGPKGAR**GAQ** **GPPGATGFPG**

901 **AAGR**VGPPGS NGNPGPPGPP GPSGKDGPKG ARGDSGPPGR AGDPGLQGPA

951 GPPGEKGEPG DDGPSGPDGP PGPQGLAGQR **GIVGLPGQR**G ER**GFPGLPGP**

1001 **SGEPGKQGAP** **GASGDRGPPG** **PVGPPGLTGP** **AGEPGR**EGSP GADGPPGRDG

1051 AAGVK**GDRGE** **TGAVGAPGAP** **GPPGSPGPAG** **PIGK**QGDR**GE** **AGAQGPMGPA**

1101 **GPAGARGMPG** **PQGPR**GDKGE TGEAGERGLK GHR**GFTGLQG** **LPGPPGPSGD**

1151 **QGASGPAGPS** **GPR**GPPGPVG PSGK**DGANGI** **PGPIGPPGPR** GRSGETGPAG

1201 PPGNPGPPGP PGPPGPGIDM SAFAGLGQRE KGPDPLQYMR ADEAAGNLRQ

1251 HDAEVDATLK SLNNQIESLR SPEGSRKNPA RTCRDLKLCH PEWKSGDYWI

1301 DPNQGCTLDA MKVFCNMETG ETCVYPNPAS VPKKNWWSSK SKDKKHIWFG

1351 ETINGGFHFS YGDDNLAPNT ANVQMTFLRL LSTEGSQNIT YHCKNSIAYL

1401 DEAAGNLKKA LLIQGSNDVE IRAEGNSRFT YTVLKDGCTK HTGKWGKTMI

1451 EYRSQKTSRL PIIDIAPMDI GGPEQEFGVD IGPVCFL

Supplementary Figure 4. Highlighted amino acid sequences identified from Capra COLIIA1 by peptide mass finger printing using *Bos taurus* COLIIA1 as template sequence.

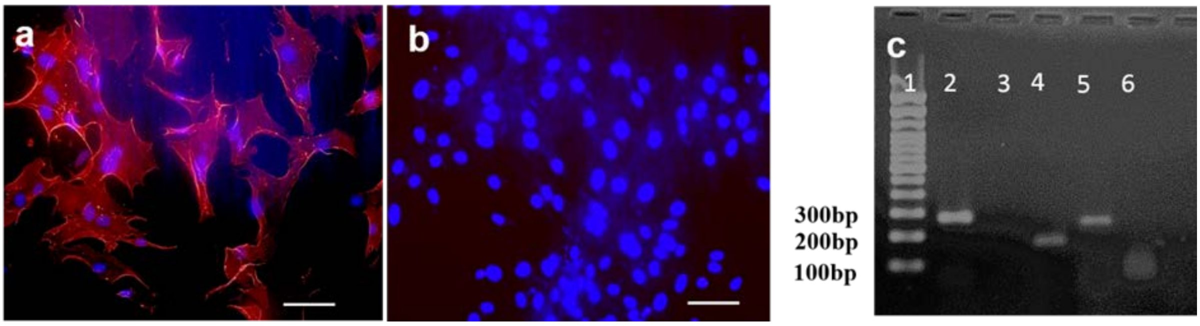

Supplementary Figure 5. Identification of isolated capra ADMSCs. Protein expression analysis showed (A) positive expression of CD 44 marker and (B) negative expression of CD 31 marker (pseudo colors, Scale bars: 100 μm); (C) RT-PCR study revealed expression of GAPDH (lane 2), CD 73 (lane 4), CD 90 (lane 5) and CD 105 (lane 6) and negative expression of CD 45 marker associated genes (lane 3) in reference to 100 bp ladder (lane 1).

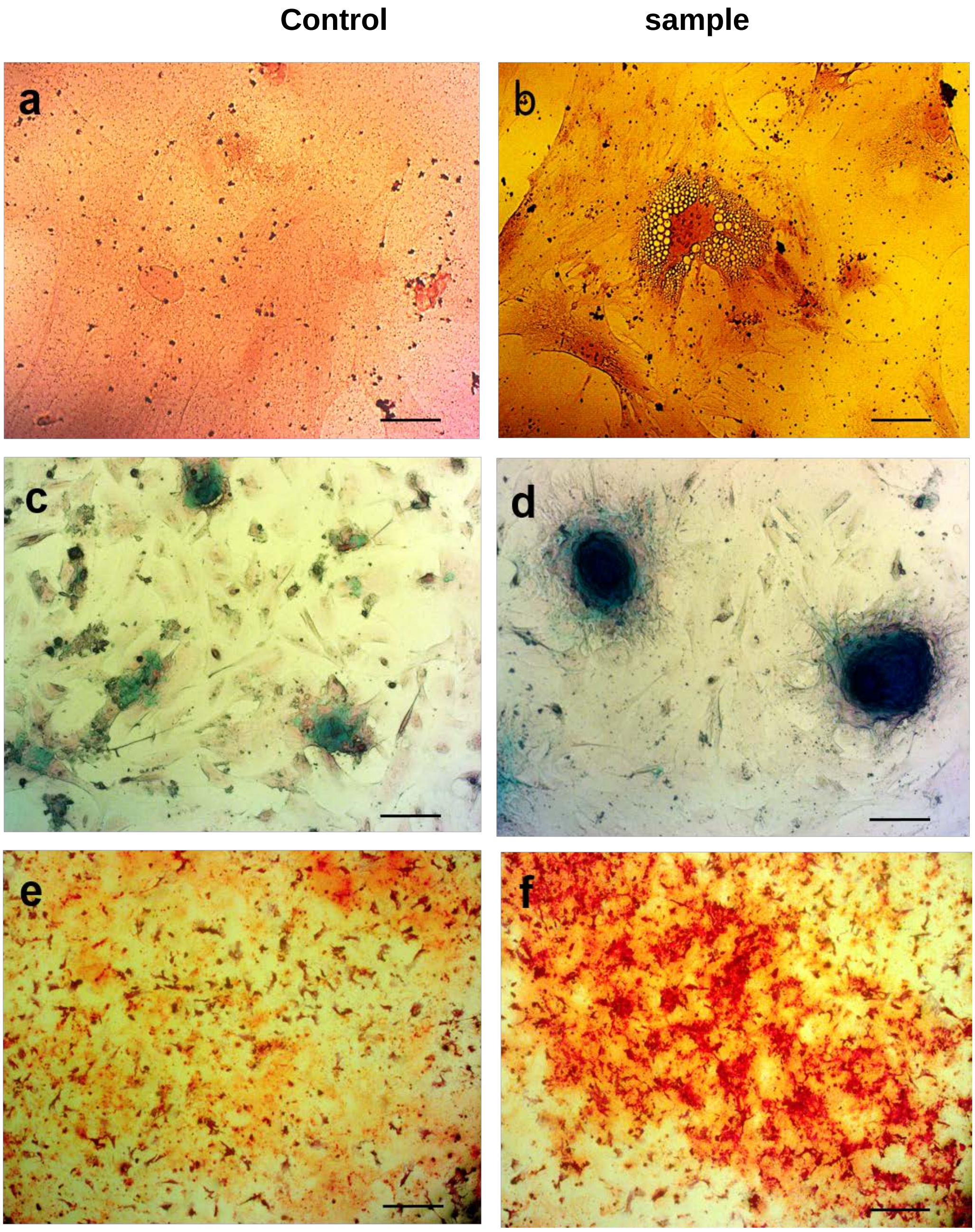

Supplementary Figure 6. Assessment of trilineage differentiation potential of ADMSCs to adipogenic, chondrogenic and osteogenic lineage. (A-B) showing significant accumulation of lipid droplets by Oil Red O staining in cytoplasm after cells cultured under adipogenic differentiation media compared to control (Scale bars: 50 μm); (C-D) depicting cartilage micro-tissue formation as stained by Alcian Blue after cells cultured under chondrogenic differentiation media compared to control (Scale bars: 100 μm); (E-F) identified significant calcium deposition by Alizarin Red S staining after cells cultured under osteogenic differentiation media as compared to control (Scale bars: 500 μm).

Supplementary Table 1: Predictive values of percentage denaturation of COL II secondary structure

**Temp. (^{o}C)**	**α-helix**	**β-strand**	**% of denature**	**% of intactness**
25	53.23	8.04	0	100
30	53.26	7.68	0	100
35	52.49	7.93	1.4	98.60
40	50.26	8.25	5.58	94.42
41	44.02	8.74	17.31	82.69
42	37.61	9.71	29.35	70.65
**43**	**25.98**	**11.68**	**51.2**	**48.80**
44	19.02	12.20	64.27	35.73
45	17.38	12.07	67.35	32.65
46	17.73	11.99	66.7	33.30
47	16.16	12.07	69.55	30.35
48	16.12	12.35	69.72	30.28
49	15.69	12.11	70.53	29.47
50	15.33	12.47	77.21	28.79

Supplementary Table 2: Comparative analysis of amino acid residues in Capra ear cartilage derived COLII with Chicken samples as reported by Cao *et al*., 2008[16]

Amino Acid	COL II (Cao *et al.*, 2008)(residues/1000 residues)	Capra ear cartilage derived COL II in present study (residues/1000 residues)
Glycine	313	302
Alanine	102	105
Glutamic acid	94	77
Proline	94	99
Hydroxyproline	118	118
Arginine	53	55
Leucine	31	29
Histidine	4	5
Lysine	15	19
Phenylalanine	15	21
Isoleucine	13	14
Methionine	2	10
Valine	22	25
Serine	25	27
Threanine	30	28
Aspartic Acid	47	41
Tyrosine	5	5
Cysteine	17	20

Supplementary Table 3: Comparisons of COLIIA1 MASCOT score with other proteins

Protein	Mass	Score
COLIIA1_Bos taurus	134858	82
COLIIA1_Bos mutus	134842	82
COLIIA1_Bos indicus	142919	73
COLIIA1_Pantholops hodgsonii	134852	76
COLIIA1_Bubalus bubalis	134844	76
COLIIA1_Homo sapiens	142782	56

Supplementary Table 4: HPLC solvent system for amino acid analysis

A-Buffer (0.01M Na_2HPO_4); B- Acetonitrile, Excitation:230, Emission: 460						
**Time**	**A%**	**B%**	**C%**	**D%**	**Flow rate ml/min**	**Max. Pressure limit (Bar)**
0.00	91.0	9.0	0.0	0.0	1.000	400.00
2.00	80.0	20.0	0.0	0.0	1.000	400.00
35.00	68.0	32.0	0.0	0.0	1.000	400.00
55.00	20.0	80.0	0.0	0.0	1.000	400.00
57.00	91.0	9.0	0.0	0.0	1.000	400.00

Supplementary Table 5: Designed Primers for RT-PCR

Genes	Sequence	Annealing Temp.(ºC)	Product Size (bp)	References
GAPDH-FP	*GGCGCTGCCAAGGCCGTGGGCAAG*	73.0	273	KEGG_100860743
GAPDH-RP	*GGCAATGCCAGCCCCAGCATCGAAGG*			
CD90 -FP	*GCACCATGAACCCTACCATC*	55	241	Mohamad-Fauzi et al., 2015 [22]
CD90 -RP	*TTGGTTCGGGAGCTGTATTC*			
CD73-FP	*CTGAGACACCCGGATGAGAT*	52	160	
CD73-RP	*ACTGGACCAGGTCAAAGGTG*			
CD105-FP	*AGATGCCAACATCACACAGC*	49	129	
CD105-RP	*TCCAGACGAAGGAAGATGCT*			
CD45-FP	*GGGAGGAGGGAAAGCAAACC*	55	146	
CD45-RP	*GCAGCTCTTCCCCATTCCAG*			
COLIIA1-FP	*GGCGCTGCCAAGGCCGTGGGCAAG*	64	269	KEGG_100860743
OLIIA1-RP	*GGCAATGCCAGCCCCAGCATCGAAGG*			
